# Supplementary figures and images for: Extracellular tau clearance is governed by its aggregation state and independent of microglial activation by LPS and IFN-γ
Source: bioRxiv. 2025 Aug 10:2025.06.23.661190. Originally published 2025 Jun 25. Preprint. [Version 2] doi: 10.1101/2025.06.23.661190 (PMC12262714; doi:10.1101/2025.06.23.661190)

**Figure S1E**

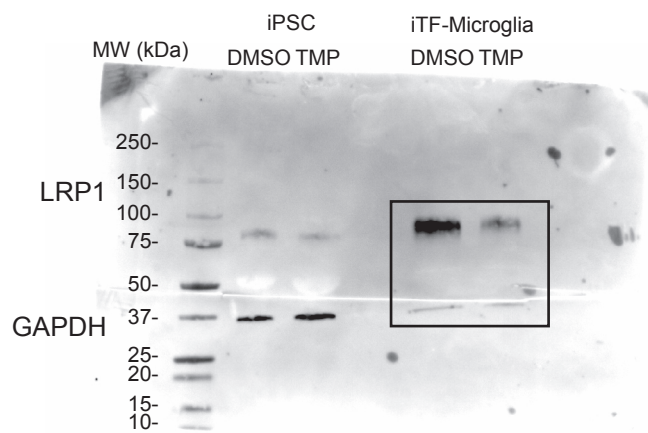

**Figure S1E**

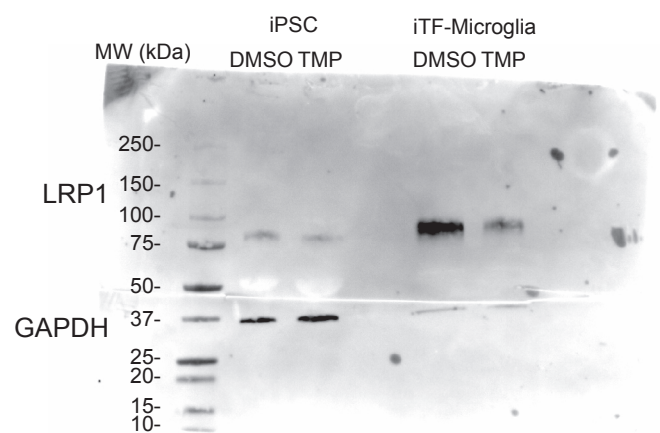

**Figure S2A**

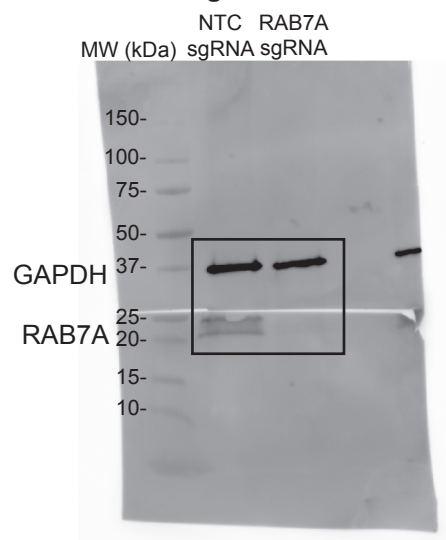

**Figure S2A**

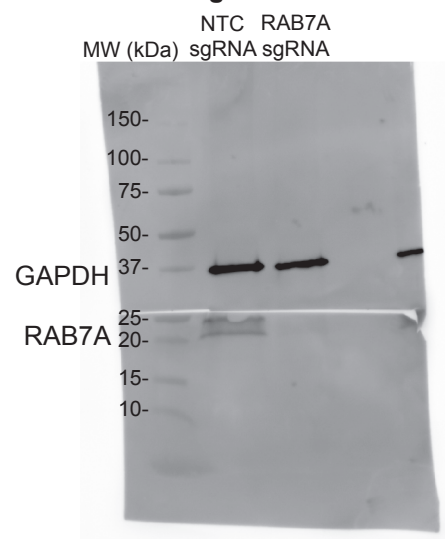

**Figure S3A**

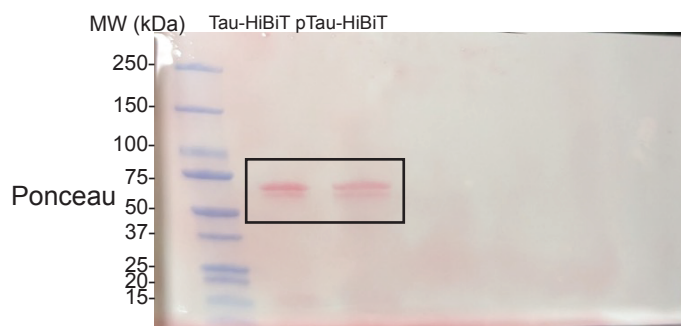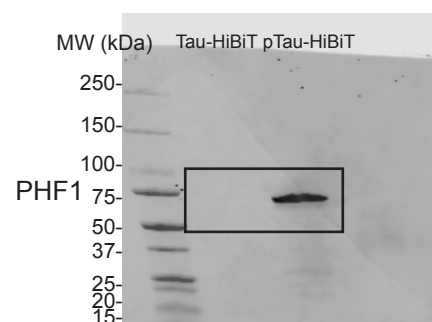

**Figure S3A**

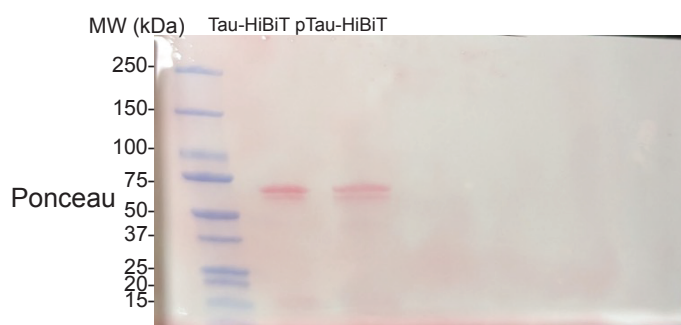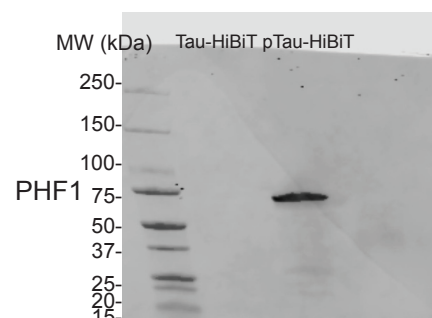

Supplement: Supplement 1 — Images provided of uncropped western blots for Figure S1, Figure S2B, and Figure S3A. [file media-1.pdf]

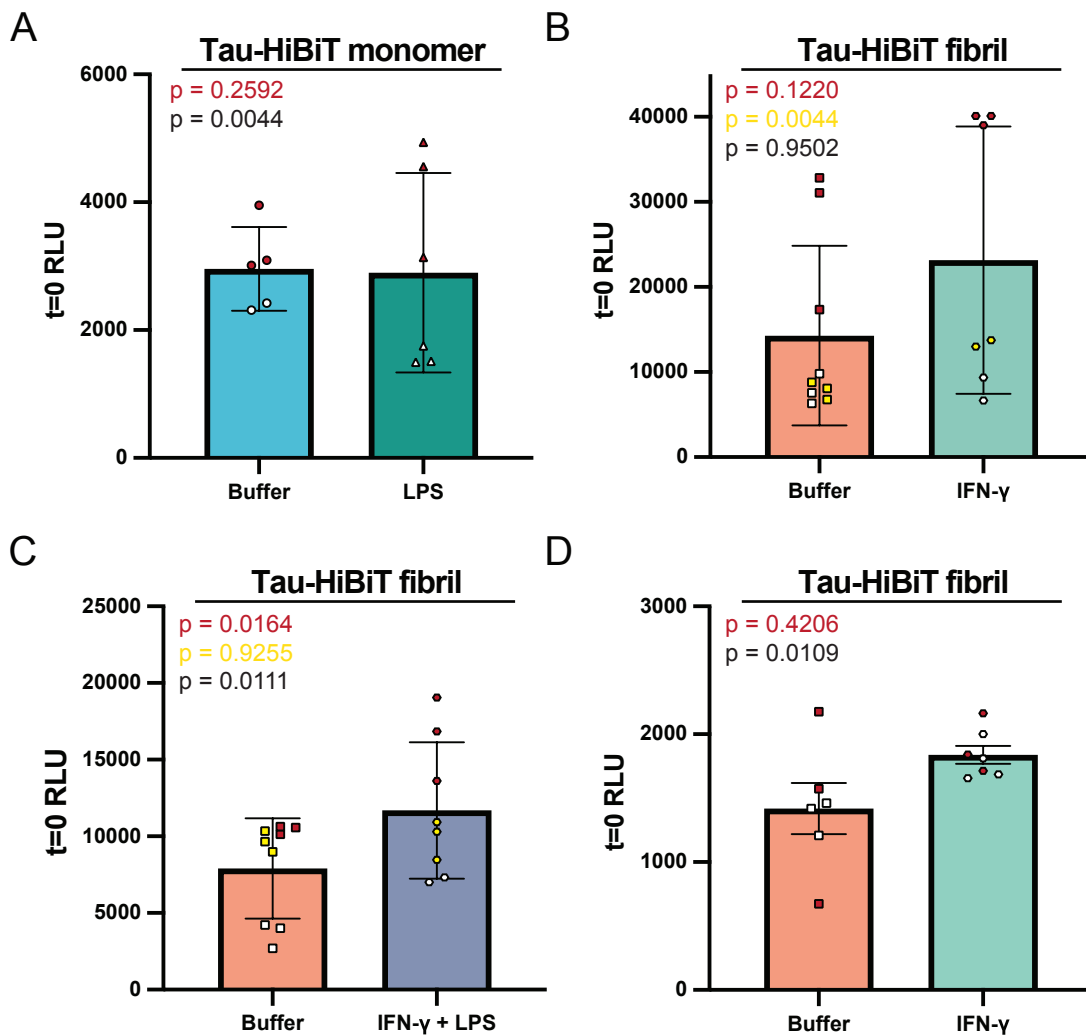

Supplement: Supplement 2 — (A) Raw t=0 RLU data corresponding to normalized data presented in Figure 5A. (B) Raw t=0 RLU data corresponding to normalized data presented in Figure 5D. (C) Raw t=0 RLU data corresponding to normalized data presented in Figure 5E. (D) Raw t=0 RLU data corresponding to normalized data presented in Figure 5H. For all data, biological replicates from independent differentiations are shown in differing colors and p values from unpaired Welch’s t-test for each cohort is displayed. [file media-2.pdf]

A

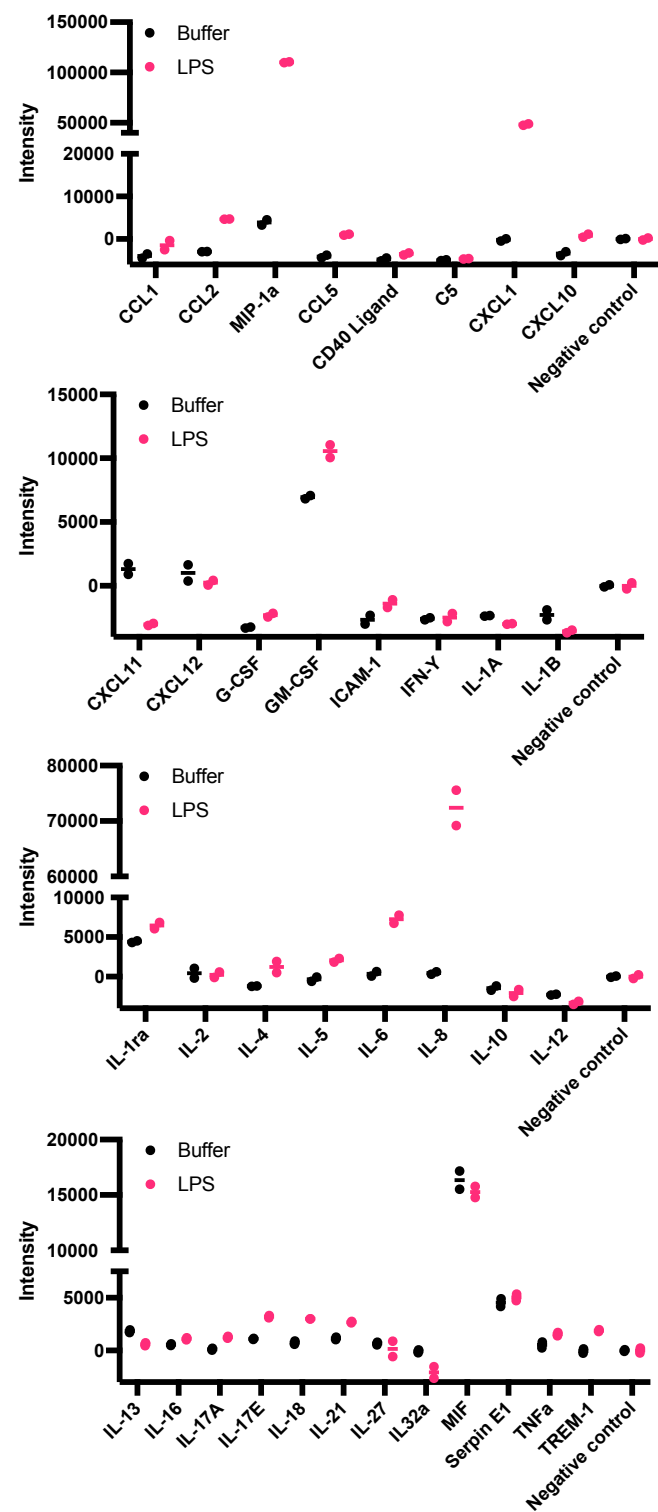

B

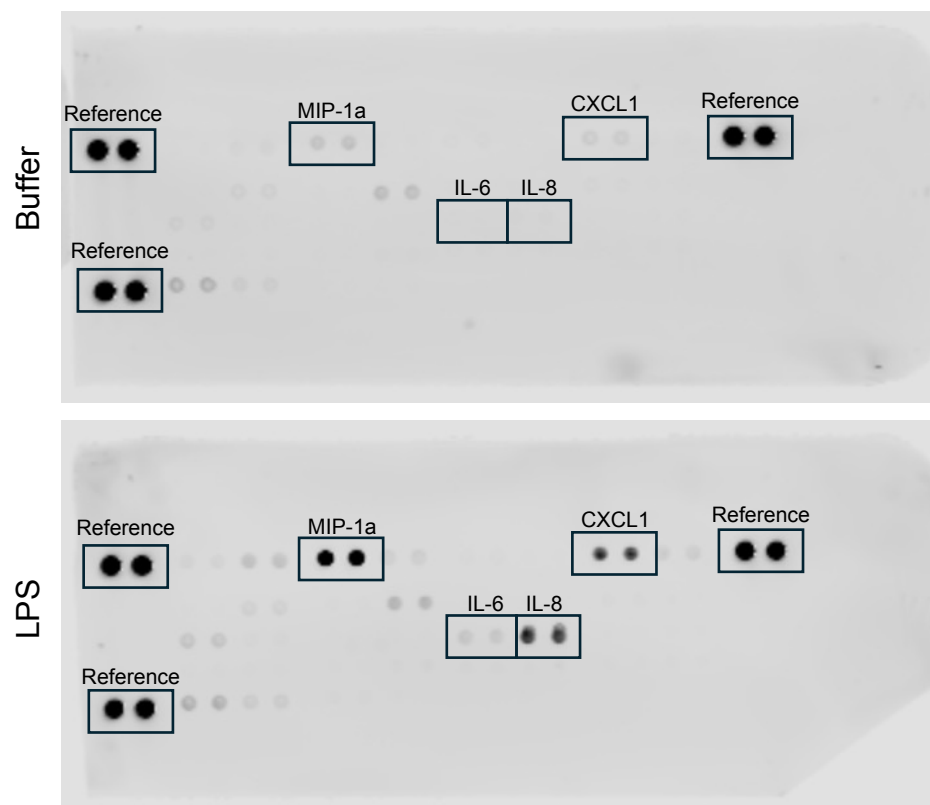

C

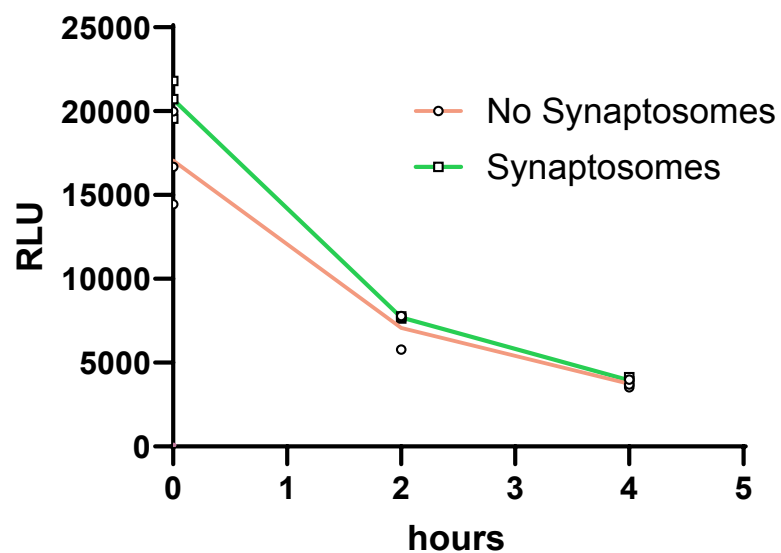

Supplement: Supplement 3 — (A) Quantification of cytokine array panel shown in (B). (B) Representative cytokine array panel. Membranes from the Proteome profiler human cytokine array kit were incubated with conditioned media from iTF-Microglia that were stimulated with Buffer (Top) or LPS (100ng/mL, 24 hours) (Bottom). (C) Clearance of fibrillized Tau-HiBiT (50nM) in iTF-Microglia treated with rat synaptosomes (0.5mg/mL) for 24 h (mean±SD; n = 3). [file media-3.pdf]

A

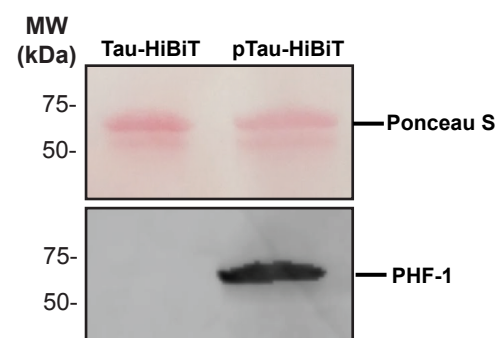

B

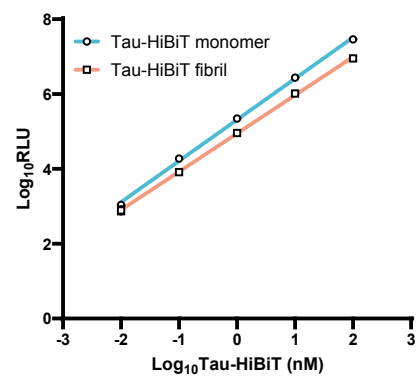

Supplement: Supplement 4 — (A) Western Blot of pTau-HiBiT protein. PHF-1 antibody recognizes pS396/pS404. (B) Luminescence of Tau-HiBiT proteins (100nM to 10pM) (mean±SD; n = 2). [file media-4.pdf]

A

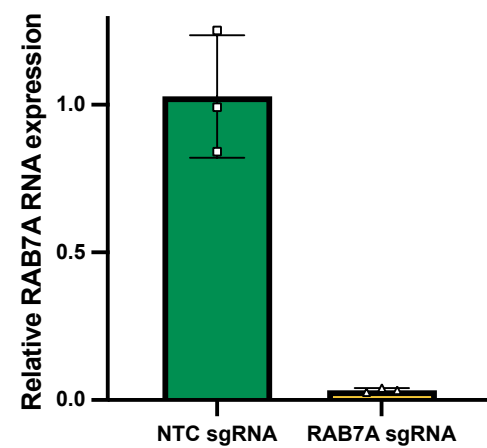

B

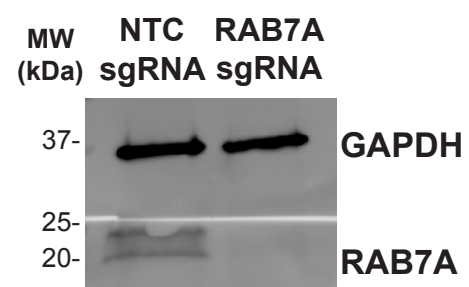

Supplement: Supplement 5 — (A) RT-qPCR of RAB7A gene expression in H4i cells. Relative gene expression was calculated using the delta Ct (ΔΔCt) with GAPDH used as a housekeeping gene (mean±SD; n = 3). (B) Western Blot of H4i cells expressing NTC sgRNA and RAB7A sgRNA. [file media-5.pdf]

A

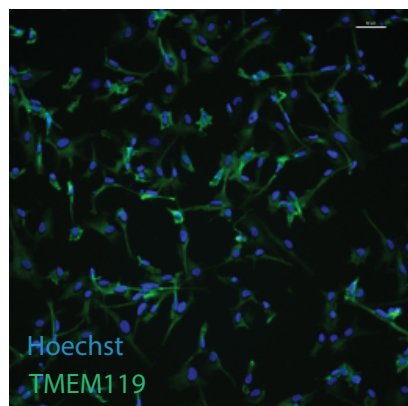

B

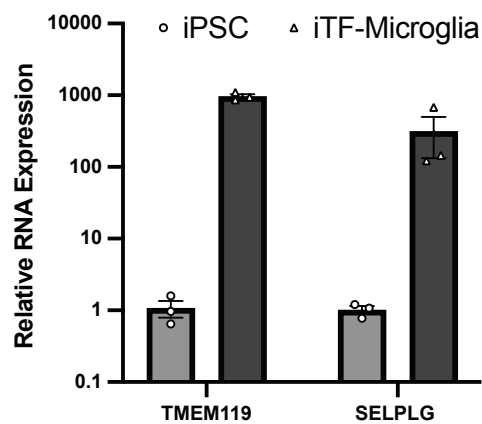

C

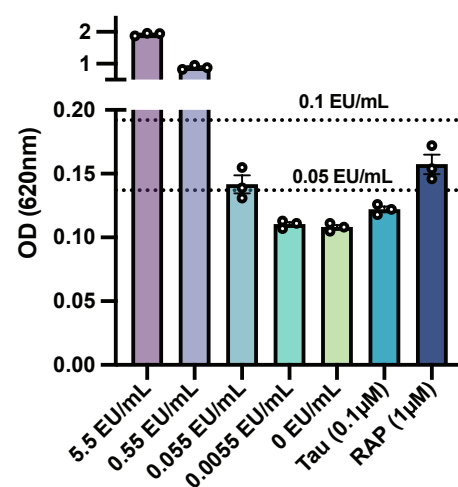

D

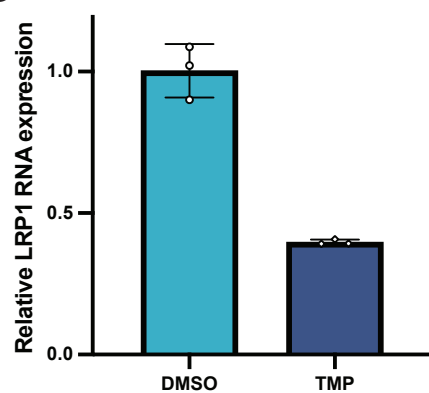

E

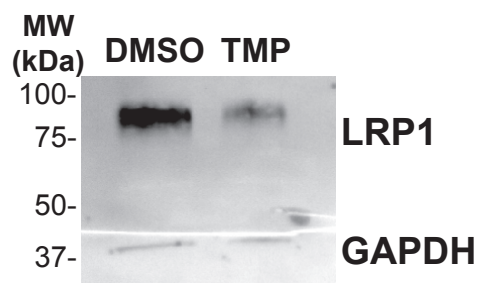

F

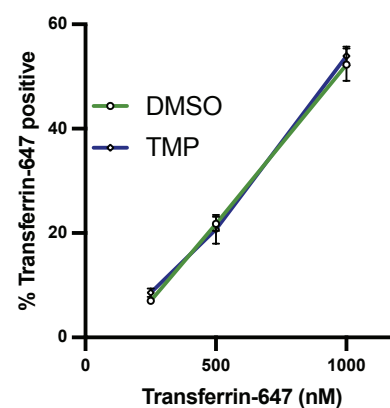

G

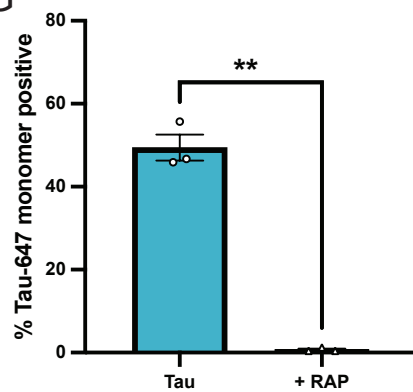

H

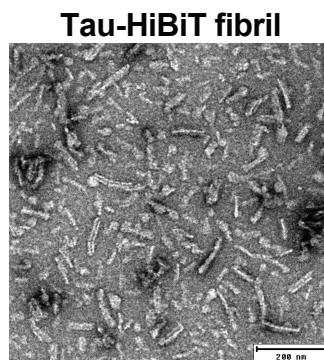

I

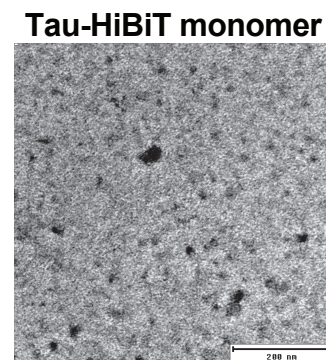

J

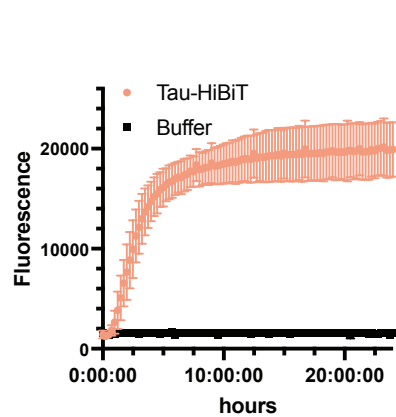

K

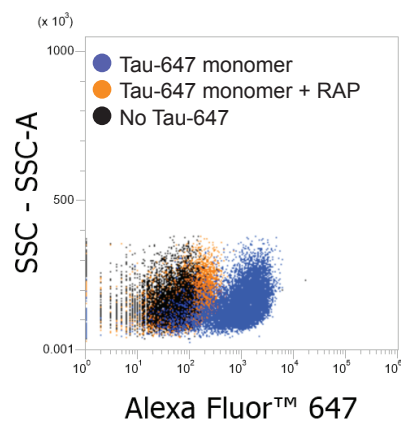

L

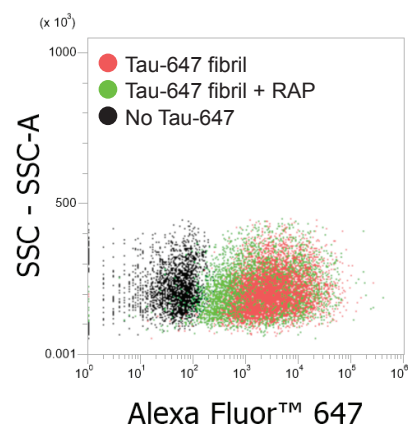

Supplement: Supplement 6 — (A) Representative ICC of iTF-Microglia after differentiation stained for TMEM119 (Green). Nuclei are stained with Hoechst (Blue); scale bar = 50μm. (B) RT-qPCR of iTF-Microglia after differentiation. Relative gene expression was calculated using the delta Ct (ΔΔCt) method with GAPDH used as a housekeeping gene; n=3. (C) HEK-Blue hTLR4 cells were incubated with endotoxin standards or recombinantly expressed proteins. Conditioned media was collected and quantified according to manufacturer’s recommendations. Calculated OD(620nm) for 0.1 EU/mL and 0.05 EU/mL are indicated with dashed-lines (mean±SD; n = 3). (D) RT-qPCR of LRP1 gene expression in iTF-Microglia with (TMP, 20μM) or without (DMSO) LRP1 knockdown. Relative gene expression was calculated using the delta Ct (ΔΔCt) method with GAPDH used as a housekeeping gene (mean±SD; n = 3 from one independent day). (E) Western Blot of iTF-Microglia with (TMP, 20μM) or without (DMSO) LRP1 knockdown. (F) Transferrin uptake in iTF-Microglia with (TMP, 20μM) or without (DMSO) LRP1 knockdown (mean±SD; n = 3). (G) Tau uptake in iMGLs incubated with monomeric Tau-647 (50nM) or Tau-647 (50nM) + RAP (250nM) for 1 h at 37°C (mean±SD; n = 3; ∗∗p < 0. 01 using unpaired Welch’s t-test) (H) TEM negative stain image of Tau-HiBit fibrils. Scale bar = 200nm. (I) TEM negative stain image of Tau-HiBiT monomer. Scale bar = 200nm. (J) ThioflavinT fluorescence measured during aggregation of Tau-HiBiT (mean±SD; n = 9). (K) Representative flow cytometry graph from iTF-Microglia after Tau-647 monomer uptake, corresponding to data in Figure 1C. (L) Representative flow cytometry graph from iTF-Microglia after Tau-647 fibril uptake, corresponding to data in Figure 1E. [file media-6.pdf]
