# Supplementary material for: Tau endo-lysosomal processing in human iPSC-derived microglia is impacted by tau aggregation state, but not by microglial activation status
Source: bioRxiv. 2025 Jun 25:2025.06.23.661190. Preprint. [Version 1] doi: 10.1101/2025.06.23.661190 (PMC12262714; doi:10.1101/2025.06.23.661190)
Supplement: Supplementary file 1 [file media-1.pdf]

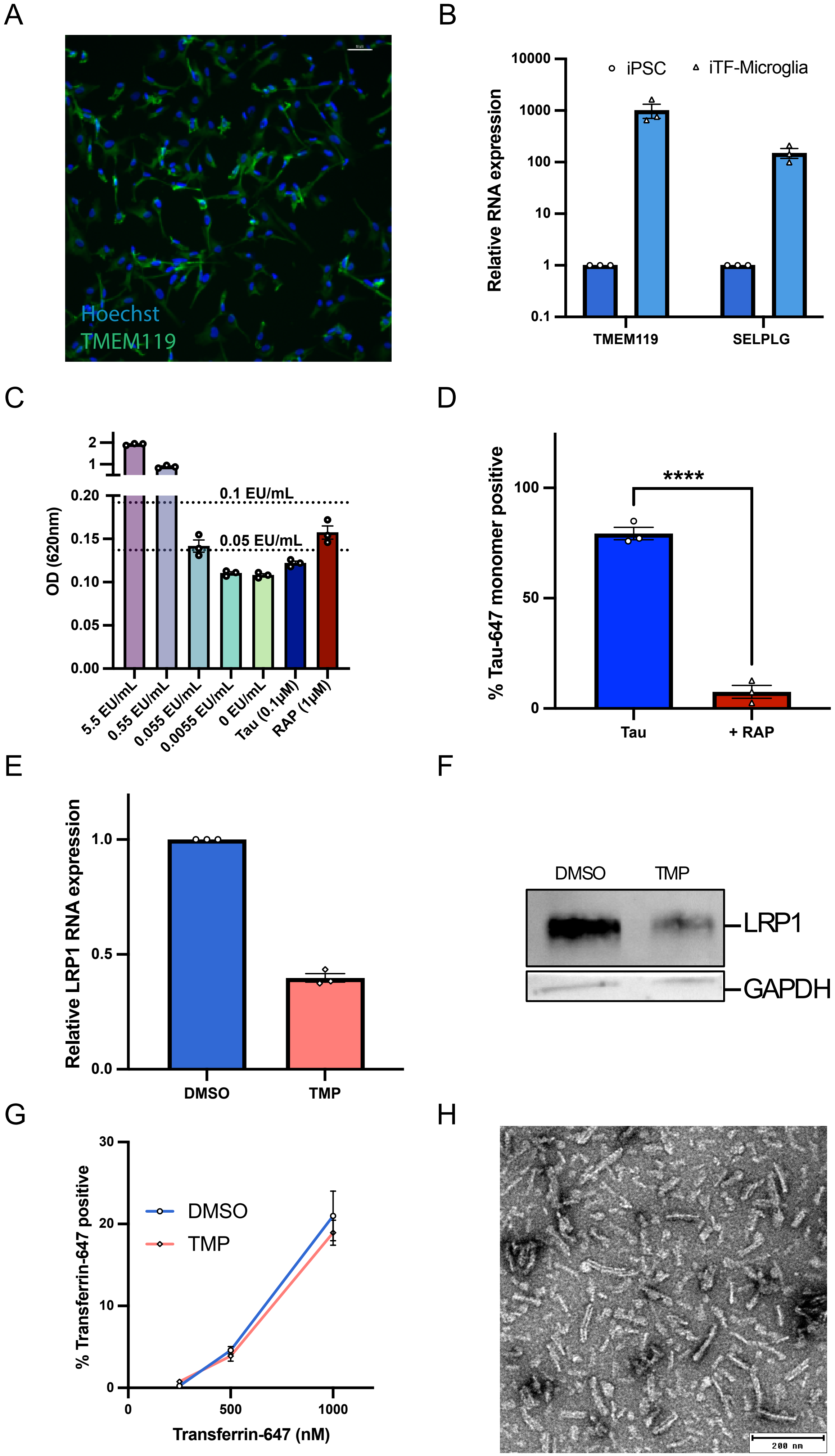
A
Hoechst
TMEM119
B
10000
1000
100
10
1
0.1
Relative RNA expression
iPSC
iTF-Microglia
TMEM119
SELPLG
C
2
1
0.20
0.15
0.10
0.05
0.00
OD (620nm)
0.1 EU/mL
0.05 EU/mL
5.5 EU/mL
0.55 EU/mL
0.055 EU/mL
0.0055 EU/mL
0 EU/mL
Tau (0.1μM)
RAP (1μM)
D
100
50
0
% Tau-647 monomer positive
****
Tau
+ RAP
E
1.0
0.5
0.0
Relative LRP1 RNA expression
DMSO
TMP
F
DMSO
TMP
LRP1
GAPDH
G
30
20
10
0
% Transferrin-647 positive
DMSO
TMP
0
500
1000
Transferrin-647 (nM)
H
200 nm
