# Supplementary material for: Tau endo-lysosomal processing in human iPSC-derived microglia is impacted by tau aggregation state, but not by microglial activation status
Source: bioRxiv. 2025 Jun 25:2025.06.23.661190. Preprint. [Version 1] doi: 10.1101/2025.06.23.661190 (PMC12262714; doi:10.1101/2025.06.23.661190)
Supplement: Supplementary file 2 [file media-2.pdf]

A

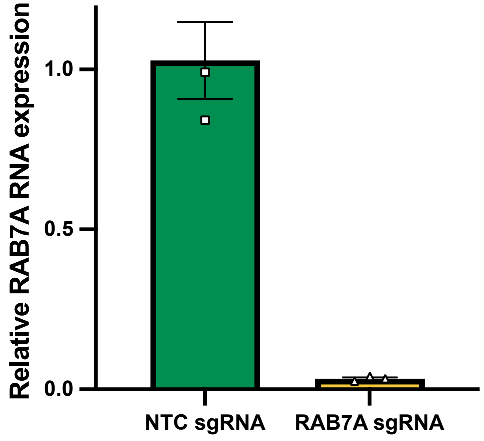
Relative RAB7A RNA expression
1.0
0.5
0.0
NTC sgRNA
RAB7A sgRNA

B

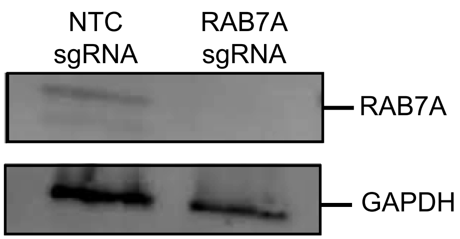
NTC sgRNA
RAB7A sgRNA
RAB7A
GAPDH
