# Supplementary material for: Tau endo-lysosomal processing in human iPSC-derived microglia is impacted by tau aggregation state, but not by microglial activation status
Source: bioRxiv. 2025 Jun 25:2025.06.23.661190. Preprint. [Version 1] doi: 10.1101/2025.06.23.661190 (PMC12262714; doi:10.1101/2025.06.23.661190)
Supplement: Supplementary file 3 [file media-3.pdf]

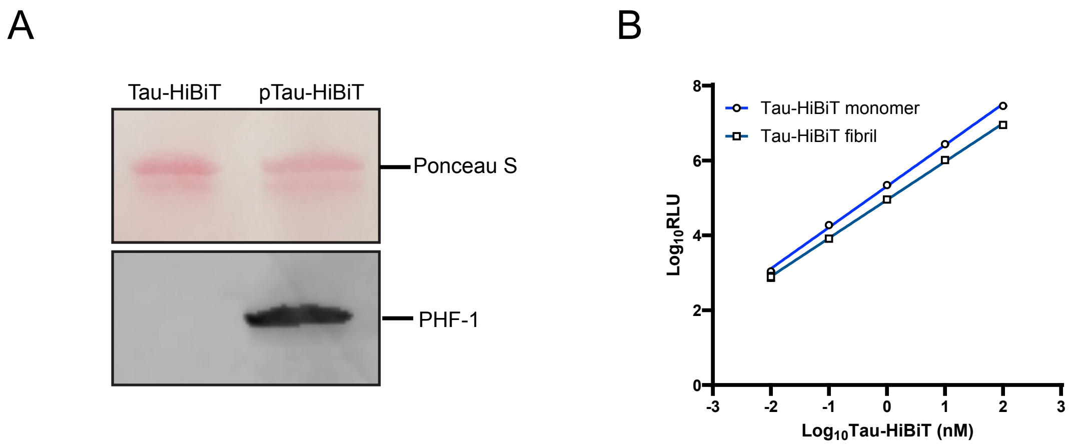
A
Tau-HiBiT
pTau-HiBiT
Ponceau S
PHF-1
B
Tau-HiBiT monomer
Tau-HiBiT fibril
Log10RLU
Log10Tau-HiBiT (nM)
8
6
4
2
0
-3
-2
-1
0
1
2
3
