# Supplementary material for: Tau endo-lysosomal processing in human iPSC-derived microglia is impacted by tau aggregation state, but not by microglial activation status
Source: bioRxiv. 2025 Jun 25:2025.06.23.661190. Preprint. [Version 1] doi: 10.1101/2025.06.23.661190 (PMC12262714; doi:10.1101/2025.06.23.661190)
Supplement: Supplementary file 4 [file media-4.pdf]

A

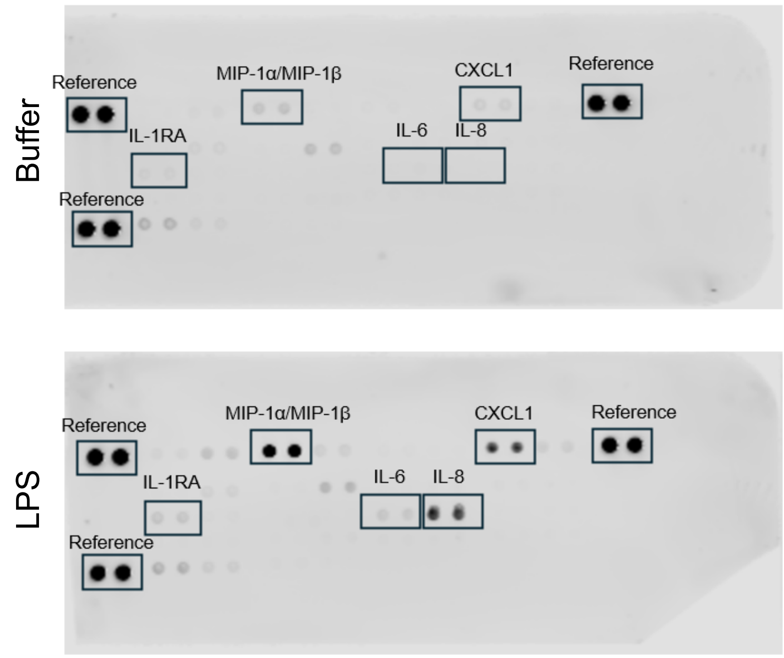
Buffer
Reference
MIP-1α/MIP-1β
CXCL1
Reference
IL-1RA
IL-6
IL-8
Reference
LPS
Reference
MIP-1α/MIP-1β
CXCL1
Reference
IL-1RA
IL-6
IL-8
Reference

B

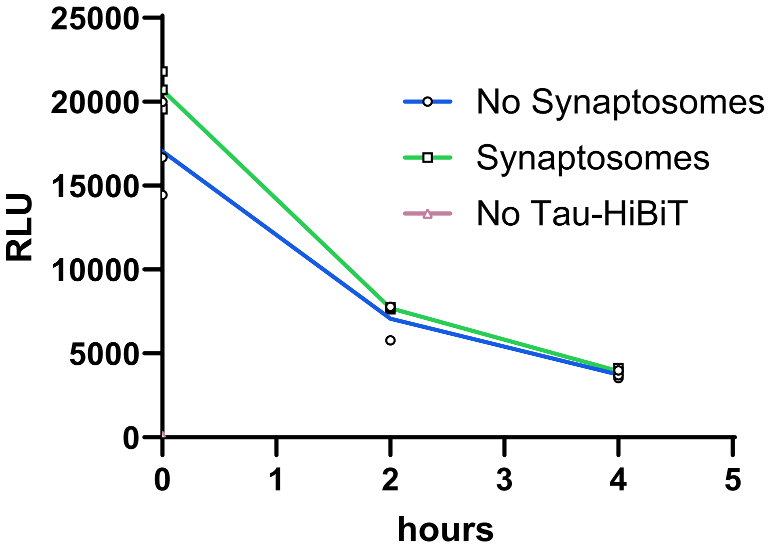
RLU
25000
20000
15000
10000
5000
0
0
1
2
3
4
5
hours
No Synaptosomes
Synaptosomes
No Tau-HiBiT
